# Supplementary material for: Evidence supported by Mendelian randomization: impact on inflammatory factors in knee osteoarthritis
Source: Front Med (Lausanne). 2024 May 28;11:1382836. doi: 10.3389/fmed.2024.1382836 (PMC11165061; doi:10.3389/fmed.2024.1382836)
Supplement: Supplementary file 1 [file Data_Sheet_1.ZIP › data/GCST90274799_5e6/799.leaveoneout.pdf]

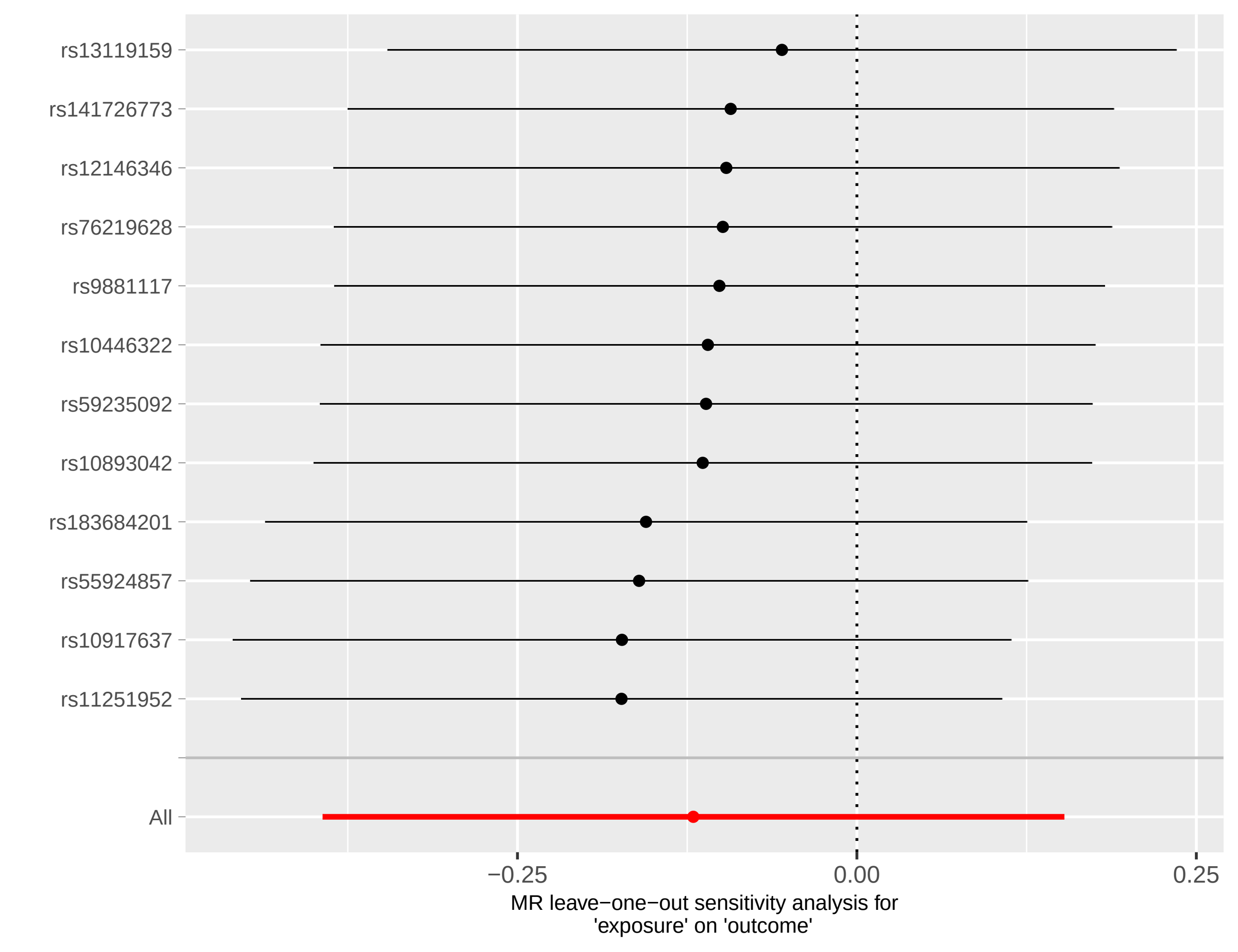

rs13119159
rs141726773
rs12146346
rs76219628
rs9881117
rs10446322
rs59235092
rs10893042
rs183684201
rs55924857
rs10917637
rs11251952
All
−0.25
0.00
0.25
MR leave−one−out sensitivity analysis for
'exposure' on 'outcome'
